# Marrow Adipose Tissue: Its Origin, Function, and Regulation in Bone Remodeling and Regeneration

**DOI:** 10.1155/2018/7098456

**Published:** 2018-05-31

**Authors:** Qiwen Li, Yunshu Wu, Ning Kang

**Affiliations:** ^1^State Key Laboratory of Oral Diseases, National Clinical Research Centre for Oral Diseases, West China Hospital of Stomatology, Sichuan University, Chengdu 610041, China; ^2^Department of Oral Implantology, West China Hospital of Stomatology, Sichuan University, Chengdu 610041, China

## Abstract

Marrow adipose tissue (MAT) is a unique fat depot in the bone marrow and exhibits close relationship with hematopoiesis and bone homeostasis. MAT is distinct from peripheral adipose tissue in respect of its heterogeneous origin, site-specific distribution, and complex and perplexing function. Though MAT is indicated to function in hematopoiesis, skeletal remodeling, and energy metabolism, its explicit characterization still requires further research. In this review, we highlight recent advancement made in MAT regarding the origin and distribution of MAT, the local interaction with bone homeostasis and hematopoietic niche, the systemic endocrine regulation of metabolism, and MAT-based strategies to enhance bone formation.

## 1. Introduction

Bone is a dynamic organ undergoing constant remodeling, where bone formation and resorption are tightly controlled by local signals and systemic cues [1, 2]. The bone marrow (BM) compartment provides a local microenvironment crucial for bone remodeling [3]. BM is composed of heterogeneous components including marrow stroma, blood vessel, progenitors of hematopoietic and skeletal lineages, and fibroblasts. The development of bone and BM is accompanied by active hematopoiesis and osteogenesis [3]. However, the formation and expansion of MAT are often observed, as a consequence of impaired hematopoiesis and osteogenesis with aging or under pathological conditions such as osteoporosis [4]. Previously, MAT was merely considered a space filler of the BM with unknown origin and function. This view persisted until 1992 when Beresford et al. proposed the opinion that osteoblasts and adipocytes share the same progenitors, known as bone marrow mesenchymal stromal cells (BMSCs) [5]. As research goes deeper, as well as the employment of novel experiment techniques such as lineage tracing, it is now acknowledged that MAT originates from skeletal lineages, functioning crucially in bone and bone marrow homeostasis, and is associated with systemic energy metabolism [3, 4]. In this review, we present a detailed discussion regarding the origin, distribution, and function of MAT, based on its difference from peripheral adipose tissue, its relationship with mesenchymal stem cells and hematopoietic niche, and its endocrine function. Moreover, regulating signals of MAT and bone remodeling, as well as strategies targeting MAT to promote bone regeneration, will also be discussed.

## 2. Development of MAT

### 2.1. MAT Is Different from Peripheral/Extramedullary Adipose Tissue

#### 2.1.1. MAT Is Located Differently

Based on their location, origin, characterization, and function, adipose tissues are typically classified into white adipose tissue (WAT), brown adipose tissue (BAT), beige adipose tissue (beige AT), and marrow adipose tissue (MAT) [6–8]. These tissues are distributed differently throughout the body. WAT depots make up most adipose tissues and are widely dispersed subcutaneously (SWAT; in the buttocks, thighs, and abdomen, accounting for 85% of the total adipose tissues) and viscerally (VWAT; around the omentum, intestines, and perirenal areas, 10%) [9–11]. BAT is much less than WAT and mainly resides in cervical, axillary, interscapular, and supraclavicular regions [11, 12]. Beige AT, however, is interspersed within (inguinal) WAT rather than residing discretely [13, 14]. MAT is found in the ribs, sternum, vertebrae, and medullary canal of long bones (tibia, femur, and humerus) [15]. In 1976, Tavassoli identified two histochemically distinct types of MAT by performic acid-Schiff staining (PFAS). Adipocytes of the red marrow were stained positively while those of the yellow marrow were PFAS-negative [16]. In the red marrow where hematopoiesis and bone remodeling are active, adipocytes are less frequent and account for up to 45% of the marrow, while in the yellow marrow where hematopoiesis is almost absent, adipocytes are densely packed and fill up to 90% of the marrow compartment [17]. A recent study further confirmed the developmental and histological difference of these two types of MAT in the red marrow and yellow marrow, which were, respectively, termed as regulated MAT (rMAT) and constitutive MAT (cMAT) [15]. rMAT is interspersed within active hematopoietic sites such as mid- to proximal tibia, femur, and lumbar vertebrae whereas cMAT is located at the distal tibia and caudal vertebrae of the tail [15].

The difference between rMAT and cMAT is more than space occupancy. Temporally, rMAT develops throughout life and is strain-specific, while cMAT develops rapidly after birth. rMAT adipocytes are smaller (32.5 ± 2.4 *μ*m diameter) than cMAT adipocytes (37.8 ± 1.2 *μ*m diameter) in C3H mice [15]. Moreover, compared with cMAT adipocytes, rMAT adipocytes contain more saturated fatty acids which are similar to WAT. When exposed to cold for 3 weeks, rMAT decreased in volume whereas cMAT was highly resistant and stable. Knockout of *ptrf* in mice could induce type 4 congenital generalized lipodystrophy (CGL4) which showed nearly complete loss of rMAT while cMAT was hardly affected. The gene expression profile of rMAT and cMAT showed that peroxisome proliferated-activated receptor gamma (PPAR*γ*) expression level paralleled but CAAT enhancer-binding protein *α* and *β* (Cebp*α* and Cebp*β*) levels were lower in rMAT adipocytes [15].

In brief summary, these data demonstrate the distinct location of MAT compared with peripheral AT and the heterogeneity of MAT exemplified by rMAT and cMAT.

#### 2.1.2. MAT Functions Differently

WAT adipocytes contain a unilocular lipid droplet and function as a lipid reservoir to store energy [18]. Besides, WAT is an endocrine organ secreting hormones such as adiponectin, leptin, resistin, and TNF-*α* [19]. BAT, on the other hand, is correlated with energy expenditure. BAT adipocytes contain a great number of mitochondria, where uncoupling protein-1 (UCP1) is enriched. UCP1 disrupts the ATP synthesis process and encourages heat generation [20]. Beige AT has mixed characteristics of WAT and BAT, which presents unilocular morphology similar to WAT but responds to cold typical of BAT [21]. MAT has mixed phenotypes resembling WAT and BAT-like or beige tissue. Marrow adipocytes resemble white adipocytes histologically but express brown adipocyte gene markers (*Prdm16*, *Dio2*, and *PGC1a*), which decrease with aging and diabetes [22]. Moreover, the gene expression profile of proximal tibia MAT is correlated with elevated beige adipocyte markers (*Ucp1*, *HoxC9*, *Prdm16*, *Tbx1*, and *Dio2*) compared with distal MAT, indicating the space-specific interaction between MAT and bone remodeling [23]. Recently, MAT was found to be an endocrine organ which secretes adiponectin, exerting systemic effects on the body during calorie restriction (CR) [24]. Noticeably, unlike the switch between WAT, BAT, and beige AT, as characterized by *prdm16*-regulated “beiging” of WAT and “whitening” of BAT, the switch between MAT and extramedullary AT has not been discovered yet [25, 26].

#### 2.1.3. MAT Originated Differently

Mature WAT adipocytes are derived from progenitors residing in the WAT depots. Using fluorescence-activated cell sorting (FACS) and Cre recombinase mouse models, Rodeheffer et al. identified and proposed an adipogenesis model where Lin^−^:CD29^+^:CD34^+^:Sca-1^+^:CD24^+^ (CD24^+^) adipocyte progenitors gave rise to Lin^−^:CD29^+^:CD34^+^:Sca-1^+^:CD24^−^ (CD24^−^) preadipocytes, which subsequently differentiated into mature adipocytes. Furthermore, lineage tracing using *PdgfRα^−^*, *Cdh5^−^*, *Tie2-cre:mT/mG* mice demonstrated that adipocytes and precursors are mesenchyme-derived instead of being endothelial- or hematopoietic-derived. Both CD24^+^ and CD24^−^ adipocyte precursors are *PdgfRα^+^* while mature adipocytes are *PdgfRα*^−^ [27]. BAT, on the other hand, is traced to myogenic lineages [28]. Seale et al. reported that BAT adipocytes arose from *Myf5^+^* myogenic precursors and PRDM16 (PRD-BF1-RIZ1 homologous domain containing 16) determined the bidirectional switch between myoblasts and BAT adipocytes *in vitro* [29]. Beige adipocytes cannot be traced with *Myf5*, indicating that they are not BAT-derived. A pioneering study showed that a subset of beige adipocyte precursors originated from subcutaneous WAT but was thermogenic upon stimulation [21]. Recently, Long et al. identified that a smooth muscle-like lineage (*Myh11*-positive) could give rise to beige adipocytes [30]. However, knowledge about beige AT still remains limited. MAT adipocytes do not share progenitors with peripheral adipocytes and are now recognized to originate from mesenchymal stem cells in the bone marrow (see detailed description below).

### 2.2. Progenitors Drive MAT Formation

The bone marrow includes a subset of nonhematopoietic, multipotent, and self-renewing progenitors capable of generating skeletal lineages associated with bone, cartilage, marrow adipose tissue, and marrow stroma [31]. These progenitors are known as skeletal stem cells (SSCs), or mesenchymal stromal cells (MSCs), contributing to bone development and bone remodeling throughout the postnatal growth and adaption process, as well as maintaining hematopoietic niches [3]. Though they are once disregarded as simply “space filling” cells and disputes concerning their origin still remain, marrow adipocytes are now acknowledged to function indispensably in bone marrow homeostasis and share precursors with osteoblasts other than extramedullary adipocytes. However, before getting a deeper inquiry into the marrow adipocytes, clarification about the definition, identification, and nomenclature of (mesenchymal or skeletal) stem cells is essential.

Detailed discrimination about mesenchymal stem cells and skeletal stem cells has been discussed previously [32]. Briefly speaking, the idea of mesenchymal stem cells was initially introduced by Cohnheim as a nonhematopoietic and mesoderm-derived population in the bone marrow [33]. Later, the original concept was extended to a common progenitor of all nonhematopoietic and nonepithelial tissues of mesoderm derivation. The criteria defining MSCs were minimized as cells adherent on plastic; cells expressing CD73, CD90, and CD105 but not expressing CD34, CD45, CD14 or CD11b, CD79a or CD19, and HLA-DR surface markers *in vitro*; and cells capable of trilineage differentiation into osteoblasts, chondrocytes, and adipocytes when induced *in vitro* [34]. This popularly accepted concept of MSCs can be confusing and sometimes misleading because it lacks *in vivo* ectopic transplantation and single-cell clonal assays, which cannot be simply inferred from *in vitro* results. On the other hand, skeletal stem cells can only be assayed in the bone marrow and are thought to be included in generally recognized BMSC population, since not all BMSCs have real multipotent and self-renewing properties [35, 36]. Skeletal stem cells (SSCs) are thus defined as self-renewing, multipotent, and skeletal lineage-committed progenitors residing in the postnatal bone marrow, capable of generating cartilage, bone, marrow stroma, and marrow adipocytes *in vivo*.

The study of the marrow adipocyte origin is performed by identifying cell surface markers *in vitro* and lineage tracing genetically engineered mice *in vivo* [3]. Isolation of cell surface markers can be done using monoclonal antibodies and flow cytometry. Lineage tracing or specific ablation experiments are performed by labeling regulatory elements or genes of interest in genetically engineered mice expressing reporters or cre recombinase [3]. Dispute about the origin of marrow adipocytes remains for decades, and the three main questions arising with time keep baffling us.

The first question lies in the relationship of marrow adipocyte and extramedullary adipocyte (white adipocytes, brown adipocytes, and beige adipocytes) origin. It was demonstrated that bone marrow adipocytes expressed *Prdm16* and *Ucp-1* in mice, genes characterized for brown adipocytes [22]. To testify if marrow adipocytes share progenitors with brown adipocytes, *Myf5-cre:mT/mG* mice were constructed. Bone marrow adipocytes were then induced with rosiglitazone, and the results showed that adipocytes were uniformly *dTomato^+^*, suggesting that the newly formed bone marrow adipocytes were not related to brown adipocyte lineage. Besides, the induced marrow adipocytes were unilocular, different from white, brown, and beige adipocytes which are multilocular [37]. Furthermore, Horowitz et al. traced adipocytes in *Osx1-Cre:mT/mG* mice. The results showed uniform dTomato^+^ cells in white and brown adipocyte tissues while eGFP^+^ adipocytes were only traced in the bone marrow [38]. Therefore, marrow adipocytes do not share the same progenitors with extramedullary adipocytes and might be derived from the bone marrow.

So the second question raised is do progenitors residing in the bone marrow give rise to marrow adipocytes. Recent studies successfully identified several markers for SSCs, including *osterix (Osx)*, *leptin receptor (LepR)*, *nestin (Nes)*, and *Gremlin1 (Grem1)* [39]. Osterix is a transcription factor crucial for osteoblast differentiation and marks different precursors at the fetal, perinatal, and adult bone stage in *Osx-*CreERT mice [40, 41]. In perinatal bone, *Osx^+^* cells were capable of trilineage differentiation *in vitro* and exhibited high CFU-F activity. Moreover, tracing perinatal *Osx^+^* cells showed a high overlap of *Osx^+^*, *nestin-*GFP^+^, and *LepR^+^* cells after several weeks, suggesting that neonatal *Osx^+^* cells are precursors of *LepR^+^* and *nestin*-GFP^+^ cells in the adult bone marrow [42]. In another experiment, Zhou et al. identified *LepR*-expressing skeletal stem cells which accounted for 0.3% of bone marrow cells while enriching 94% of CFU-Fs [43]. Besides, *LepR^+^* cells expressed PdgfR*a*, CD51, and Prx1-Cre and generate most adipocytes and osteoblasts in the adult bone marrow [43, 44]. Interestingly, most *LepR^+^* cells expressed *nestin*-GFP^low^, indicating an overlap of *LepR^+^* and *Nes^+^* SSC populations [43]. Further, a study also showed that leptin/lepR signaling promoted adipogenesis and inhibited osteogenesis in SSCs [45]. Therefore, the aforementioned results identified an *Osx^+^*, *LepR^+^*, and *Nes^+^* SSC population capable of generating adipocytes. Most recently, using a fluorescence-activated cell sorting (FACS) approach, Ambrosi et al. identified a tripotent, perivascular CD45^−^CD31^−^Sca1^+^CD24^+^ precursor which gave rise to CD45^−^CD31^−^Sca^−^PDGFR*a*^+^ osteochondrogenic progenitor cells (OPCs) and fate-committed CD45^−^CD31^−^Sca^+^CD24^−^ adipogenic progenitor cells (APCs). The latter subsequently gave rise to mature CD45^−^CD31^−^Sca1^−^zfp423^+^ preadipocytes (preAds) [46]. On the other hand, Gremlin1, the bone morphogenetic protein (BMP) antagonist, marks another SSC populations which give rise to osteoblasts, chondrocytes, myofibroblasts, and reticular marrow stromal cells but not to adipocytes [47]. Together, these studies confirm that marrow adipocytes are derived from MSCs in the bone marrow. It is also indicated that the origin of marrow adipocytes might be heterogeneous.

The third question thus lies in the definitive origin of marrow adipocytes in BM. One possible explanation is that adipocytes are derived from different progenitors in the bone marrow. This opinion is supported by the identification of different SSC subpopulations capable of adipogenic differentiation, as well as by the site-specific distribution and functional difference of MAT throughout the bone marrow, for instance, rodent rMAT and cMAT, whose origin remains unknown. However, the possibility that a single population of progenitors spatially and temporally gives rise to adipocytes cannot be excluded yet.

## 3. MAT and Bone Marrow Homeostasis

Bone marrow homeostasis, including hematopoiesis and bone remodeling, is tightly controlled by interactive communications of marrow stroma, surrounding cells, and regulatory signals at the local microenvironment. Marrow adipose tissue is considered a crucial component which influences the bone marrow homeostasis and sometimes the regeneration process. MAT alters hematopoietic stem cell (HSC) functions, contributes to the fate commitment of MSCs, and activates or inhibits osteoblasts and osteoclasts via paracrine and endocrine regulation. In this part, we focus on the effect of MAT on the bone marrow niche, as well as on the factors regulating MAT.

### 3.1. MAT and Hematopoietic Niche

It remains controversial regarding the protective or inhibitory role of MAT in hematopoiesis. Myelopoiesis and lymphopoiesis are two branches of hematopoiesis besides HSC self-renewal [48]. A series of work was carried out to study the relationship of MAT and myelopoiesis or lymphopoiesis. Adiponectin is a MAT-secreted hormone [49]. Culturing bone marrow cells with it resulted in a strong inhibitory effect on B lymphopoiesis but slightly enhanced myelopoiesis, which was mediated by the activation of prostaglandin synthesis [50]. Similarly, Bilwani et al. showed that adipocytes could secrete soluble factors that inhibit B lymphopoiesis [51]. Coculturing stromal cells (SCs), which contain a large number of adipocytes, with lineage^−^Sca-1^+^CD117^+^ (LSK) cells also greatly impaired LSK cell function [52]. A pioneering study explored the relationship between hematopoietic activity and bone marrow adiposity of different regions in mouse skeleton [53]. In the tail vertebrae where adipocytes are enriched, the HSC and progenitor activity was reduced, compared with that in the thorax vertebrae where adipocytes are bereft. The cKit^+^Lin^−^Sca1^+^Flk2^−^ HSCs, multipotent progenitors (MPPs), common myeloid progenitors (CMPs), granulocyte-macrophage progenitors (GMPs), and megakaryocyte-erythroid progenitors (MEPs) were all reduced 2-3-fold in number; meanwhile, HSCs, MPPs, and CMPs also exhibited reduced cycling in the tail vertebrae. Transwell cocultures of MAT adipocytes and hematopoietic cells exhibited congruent results. Importantly, in lipoatrophic A-ZIP/F1 mice or in PPAR*γ* inhibitor bisphenol A diglycidyl ether- (BADGE-) treated mice, MAT is inhibited but the hematopoietic recovery was rescued after lethal irradiation [53]. Recently, Yubin et al. clarified the relationship of obesity with the bone marrow niche. Mice fed with a high-fat diet (HFD) for 2 weeks exhibited reduced long-term hematopoietic LSK cells and the shift of lymphoid to myeloid cell differentiation. Besides, HFD promoted adipogenesis and inhibited osteoblastogenesis via the activation of PPAR*γ* in MSCs, leading to impaired bone architecture. Inhibiting PPAR*γ* with BADGE successfully rescued the endosteal bone niche and HSC development. In addition, gut microbiota was also found altered in HFD-treated mice and further influenced the HSC population [54]. Another study fed mice with HFD for 180 days, and the results also revealed the altered hematopoietic and lymphopoietic functions, though an enhancement effect was observed [55]. As it is introduced, Ambrosi et al. identified a development hierarchy pattern where tripotent, perivascular CD45^−^CD31^−^Sca1^+^CD24^+^ precursors gave rise to APCs and subsequent mature preAds. They, respectively, transplanted CD45^−^CD31^−^Sca1^+^CD24^+^ cells, APCs, and preAds into irradiated mice, and all these cells generated adipocytes. However, CD34^−^ long-term LSK cells and CD34^+^ short-term LSK cells were greatly reduced in APC- and preAd-transplanted group, while CD45^−^CD31^−^Sca1^+^CD24^+^ cells not only gave rise to adipocytes but also protected hematopoietic regeneration [46]. This study enlightens us that adipocyte precursors at different differentiation stages can sometimes exert distinct effect on hematopoiesis. The aforementioned studies proved the negative role of MAT in the hematopoietic niche. However, there are also some contradictory studies revealing the indispensable role of MAT in hematopoiesis. A study shows that troglitazone-induced adipocytes supported primitive hematopoietic cells *in vitro*, though no effect was observed *in vivo* [56]. Moreover, Zhou et al. discovered that MAT adipocytes arising from *Adipoq*-Cre/ER^+^ progenitors synthesized the stem cell factor (Scf), an important HSC niche factor, and promoted the hematopoietic regeneration of the mice after irradiation or 5-fluorouracil (5-FU) treatment. Adiponectin was found expressed in all MAT adipocytes, and a conditional knockout of *Scf* using *Adipoq*-Cre displayed impaired hematopoietic recovery. Interestingly, Scf from osteoblastic, hematopoietic, and endothelial cells is dispensable for hematopoietic regeneration [57]. The reason for these discrepancies is unknown, probably because of the heterogeneity of the adipocytes. Thus, future work categorizing the subpopulation of MAT adipocytes and investigating their effect on hematopoiesis based on the subpopulation is required.

### 3.2. MAT and Osteoblastogenesis/Osteoclastogenesis

Adipocytes are recognized to inhibit osteoblast activity. Coculturing human primary osteoblastic cells with human mature adipocytes resulted in inhibition of osteoblastic cell proliferation [58]. This effect is attributed to the lipotoxicity of fatty acids (FA) to osteoblasts [59]. Furthermore, it is discovered that the highest marrow adipocyte saturation was present in postmenopausal women with diabetes and fractures. This clinical trial provided evidence for the inverse relationship between MAT saturation and bone, though no association of MAT content with diabetes and fractures was indicated [60]. On the other hand, MAT adipocytes contribute to osteoclastogenesis. Bone marrow stromal cell line 2- (BMS2-) derived adipocytes were shown to support the osteoclast differentiation of primary bone marrow cells depleted of adherent stromal and macrophage populations when cocultured in vitamin D-containing microenvironment [61]. In addition, mature marrow adipocytes were capable of secreting chemokines CXCL1 and CXCL2, which promoted osteoclast maturation in metastatic prostate cancer [62]. Besides mature adipocytes, preadipocytes also contribute to osteoclastogenesis. During adipogenic differentiation of marrow stromal cells, early transcription factors, C/EBP*β* and C/EBP*δ*, bind to the receptor activator for nuclear factor-*κ*B ligand (*Rankl*) promoter and facilitate *Rankl* gene expression. Moreover, these RANKL-positive cells were positive for Pref-1, which is a preadipocyte marker. Therefore, the RANKL^+^/Pref-1^+^ cell population contributes to osteoclastogenesis [63]. Similarly, Fan et al. recently built conditional *parathyroid hormone receptor 1*- *(PTH1R*-) knockout mice using *Prx1-Cre* recombinase. The *Prx1-Cre:PTH1R^fl/fl^* mice exhibited an abnormal skeletal and marrow phenotype characterized by significantly reduced bone mass, high bone resorption, and increased MAT. They also identified an elevated number of Pref1^+^RANKL^+^ marrow progenitors in *Prx1-Cre:PTH1R^fl/fl^* mice. The results revealed that in the absence of PTH1R signaling, MSCs are prone to give rise to preadipocytes which further differentiate into MAT adipocytes and produce RANKL, leading to bone loss. Importantly, PTH treatment reduced MAT both in mice and in osteoporotic men, indicating its ability to regulate stem cell lineage allocation [64].

### 3.3. MAT as an Endocrine Organ

MAT adipocytes are capable of secreting adipokines including adiponectin and leptin [49, 65], which exert both local and systemic effects on bone and energy metabolism. Adiponectin is initially known to be derived from WAT and promotes insulin sensitivity and fat oxidation and protects against inflammation. During CR, however, MAT is markedly increased and contributes to increased circulating adiponectin [24]. A study showed that adiponectin exerts two opposite effects on bone. In short-term conditions, adiponectin inhibits osteoblast proliferation and increases their apoptosis via the PI3K-FoxO1-dependent pathway. In long-term conditions, adiponectin increases bone formation and bone mass by repressing the sympathetic nervous system [66]. Leptin is another adipokine that influences both energy and skeletal homeostases. Leptin signaling regulates bone metabolism via central and peripheral pathways. Centrally, leptin acts on LepR^+^ neurons in the hypothalamus, activating the sympathetic nervous system which favors adipogenesis [67]. Besides, leptin exerts effect on hypothalamic pituitary peripheral axes, leading to altered neuroendocrine hormones (e.g., thyroid, estrogen, cortisol, and IGF-1 hormones) which are capable of regulating bone metabolism [68–70]. Peripherally, leptin enhances bone turnover and increases bone density [70–73]. In leptin-deficient (*ob/ob*) and leptin-receptor-deficient (*db/db*) mice, impaired bone formation was observed [71–73]. Subcutaneous injection of leptin rescued the bone phenotype in leptin-deficient (*ob/ob*) mice [73]. Moreover, a series of clinical trials demonstrate the positive association between leptin and bone mass [69, 70, 74, 75]. For instance, recombinant leptin therapy increased IGF-1, osteocalcin, and alkaline phosphatase level in hypothalamic amenorrheic women [74]. In hypoleptinemic women, bone mineral density and content at the lumbar spine are increased after long-term metreleptin (synthetic leptin analog) treatment [75]. Despite these persuasive pieces of evidence, however, a recent study shows that in the bone marrow, leptin binds to LepR and the leptin/lepR signaling promotes adipogenesis and inhibits osteogenesis of SSCs. Besides, activation of LepR retarded bone healing in femur fractures or after irradiation in mice [45].

### 3.4. MAT Bridges Energy and Bone Metabolism

Anomalies in energy metabolism including anorexia nervosa (AN) in human or CR in mice, type 1 and type 2 diabetes mellitus (T1DM and T2DM), and gonadal deficiency, as well as obesity and aging, led to an alteration in MAT and bone phenotype [76]. Thus, it is apparent that there is a reciprocal regulation between bone homeostasis and energy metabolism, where MAT is simultaneously involved in both processes though no direct evidence supports the causal relationship between MAT and bone pathology. The coupled effect of skeletal and energy metabolism lies in shared hormonal signals such as insulin, adiponectin, leptin, osteocalcin, and lipocalin 2, as well as in PPAR*γ*-regulated molecular response.

AN in human or CR in mice led to a decrease in peripheral adipose tissue which was compensated for energy catabolism but paradoxically an increase in marrow adipocytes [77, 78]. The reason for this phenomenon is unclear. Besides, the patients/mice also experienced decreased bone mineral density and elevated fracture risk [78]. In patients with AN, MAT is elevated and positively correlates with circulating adiponectin and Pref-1 [24, 79]. In young CR mice, besides elevated MAT, decreased serum level of leptin and IGF-1 was observed [78]. These adipokines/hormones have been shown to regulate bone homeostasis [45, 80–82]. Nevertheless, whether MAT directly affects these adipokines/hormones and consequently influences bone mineral density requires further elucidation in patients with AN. Apart from AN, increased MAT and decreased bone mineral density are also observed with aging and gonadal deficiency [83–85]. In contrast, the relationship of obesity, MAT, and bone has not reached a consensus. Both animal experiments and clinical trials have reported various and conflicting results [60, 77, 86–90]. For instance, HFD-induced obesity in C57BL/6J mice displayed increased MAT volume, but the trabecular and cortical compartments were reported to be unchanged or impaired [91, 92]. Patients suffering from T1DM often experience bone loss and higher risk of fractures [93, 94]. However, no correlation between marrow adiposity and T1DM was observed, and marrow adiposity turned out to be linked to elevated serum lipid [95]. Moreover, harnessing the PPAR*γ* antagonist BADGE to T1DM mice reduced marrow adiposity but failed to rescue T1DM-induced bone loss [96]. The presence of various results is reasonable, because diabetes mellitus is a systemic disease with multifunctional and multiorganic disorganization, which is controlled by multifactors. Thus, more research regarding the direct role of MAT in bone pathology in consideration of different settings (e.g., gender, age, and systemic complications) of diabetes is needed.

## 4. Targeting MAT to Promote Skeletal Regeneration

### 4.1. Targeting Skeletal Stem Cells

Inducing a large number of MAT is often required in research. A high-fat diet, chemotherapy, and irradiation could lead to MAT accumulation in the bone marrow. Especially for lethal irradiation of mice, the marrow was ablated first and then intravenously or intratibially transplanted with bone marrow cells to induce MAT formation. Three days postirradiation, regenerated adipocytes could be observed in the bone marrow. This technique allows us to do lineage tracing and to test differentiation ability of cell subpopulation *in vivo*. Using this method, LepR^+^ skeletal stem cells were shown to form osteoblasts and adipocytes after irradiation. Besides, it was discovered that LepR^+^ cells were the main source of adipocytes in the adult bone marrow [43]. Therefore, targeting LepR^+^ cells and their descendants towards osteogenesis instead of adipogenesis seems to be more efficient than targeting BMSCs. The same was true for CD45^−^CD31^−^Sca^+^CD24^−^ adipogenic progenitor cells and mature CD45^−^CD31^−^Sca1^−^zfp423^+^ preadipocytes, which were found to inhibit bone regeneration of tibia fracture [46]. Interestingly, Yue et al. further tested the function of the leptin receptor in SSCs using conditionally deleted *LepR* from limb BMSCs using *Prx1*-Cre recombinase and surprisingly found out that loss of *LepR* inhibited adipogenesis and accelerated fracture healing [45]. Moreover, *PTH1R* deletion in mesenchymal stem cells leads to the formation of adipocytes, which expressed RANKL, leading to osteoclastogenesis. Therefore, receptors expressed in skeletal stem cells such as LepR and PTH1R are the potential target for skeletal regeneration [64].

### 4.2. Targeting Secreted Cytokines/Adipokines

Fatty acids can exert lipotoxic effect on primary osteoblastic cells and inhibit osteogenesis [56]. It is also found that circulating lipid level is positively correlated with MAT in diabetes. Therefore, serum lipids might be the potential target to decrease MAT [95, 97]. Low-density lipoprotein receptor-related protein 5 (LRP5) is a Wnt coreceptor which inhibits adipogenesis and promotes osteogenesis. Besides, LRP5 modifies energy metabolism by positively affecting insulin signaling [98]. Interestingly, LRP5 showed depot-specific expression in WAT, higher in abdominal than in gluteal fat progenitors, and regulated a depot-dependent Wnt/*β*-catenin pathway signaling. In addition, under equivalent knockdown of LRP5, an impaired gluteal but enhanced abdominal adipogenesis was observed. This study enlightens us that a dose- and depot-specific gene expression and signaling manner might also exist in MAT, and investigation into cMAT and rMAT can be harnessed based on this presumption [99].

Additionally, cytokines regulate marrow adipocytes in a paracrine or endocrine manner. Adiponectin and leptin, as discussed previously, are adipokines secreted by MAT which affect bone homeostasis via local and systemic regulation [45, 66]. Moreover, adiponectin decreased the level of stromal cell-derived factor 1 (SDF-1) in the bone marrow while increasing it in circulation, thereby creating a chemotactic signal crucial for BMSC mobilization and recruitment from the bone marrow to bone fractured sites [100]. Sclerostin is an osteocyte-derived glycoprotein and acts as an LRP5 antagonist, which inhibits osteogenesis and promotes adipogenesis [101]. Circulating sclerostin was found positively correlated with vertebral MAT in older men [102]. As a new drug (romosozumab) which binds to sclerostin to treat osteoporosis in postmenopausal women is about to go on sale, its potential effects on fat distribution and energy metabolism should also be noted [103]. Dipeptidyl peptidase-4 (Dpp4) is a cell surface serine protease known as an important target of diabetes treatment, as well as a secreted factor released from adipogenic cells which is negatively correlated with bone mass [104, 105]. Importantly, Ambrosi et al. have shown that CD45^−^CD31^−^Sca1^+^CD24^+^ cells and APCs in MAT can secret Dpp4. Treatment of CD45^−^CD31^−^Sca1^+^CD24^+^ cells and OPCs with the Dpp4 inhibitor sitagliptin significantly enhanced osteogenesis *in vitro* and in bone fractured sites [46]. Besides, Dpp4 is a negative regulator of colony-stimulating factor (CSF) activity and hematopoiesis in the bone marrow. Dpp4 inhibition enhanced hematopoiesis in mice [106]. Steroid hormones, including testosterone, estrogen, and glucocorticoid, regulate MAT and bone homeostasis. Both testosterone and estrogen inhibited MAT formation, as evidenced by an animal experiment and clinical trial [23, 85, 107, 108]. Glucocorticoid (GC), however, induces MAT formation and expansion. Exogenous GC treatment to patients with systemic lupus erythematosus resulted in a dose-dependent increase of MAT in the proximal femur. Besides, higher MAT was correlated with ischemic bone lesions [109]. Moreover, activation of GC in cells required 11beta-hydroxysteroid dehydrogenases (11betaHSD1). Mice deficient in 11betaHSD1 (HSD1^−/−^) exhibited a total absence of MAT adipocytes, suggesting that active GC is required for MAT formation. However, no significant change of bone mass was observed in HSD1^−/−^ mice [110].

### 4.3. Targeting Transcription Factor

Transcription factors play a crucial role in the fate commitment of MSCs [111, 112]. PPAR*γ* is a key transcription factor for the adipogenic differentiation of MSCs and differentiated state maintenance. The C/EBP family is also involved in the regulation of adipogenesis. Apart from them, transcription factors including CREB, Krox20, KLF5, and STAT5 participate in the complex network of transcription regulation [113]. Thus, antagonists targeting these factors might be utilized to decrease marrow adiposity and increase bone mass and quality. For instance, BADGE was able to induce bone formation and decreased marrow adiposity without altering blood glucose, parathyroid hormone, and Ca^2+^ levels in serum, which could be a promising drug for osteoporosis [114]. On the other hand, PPAR*γ* agonists promote adipogenesis and inhibit osteogenesis. More importantly, they could increase insulin sensitivity and regulate lipid metabolism, serving as ideal pharmaceuticals for diabetes mellitus. For instance, thiazolidinediones (TZD) are routinely used in clinical practice of T2DM treatment. Inevitably, the side effect of TZD is associated with osteoporosis and increased risk of bone fractures. Also, MAT volume is increased with TZD administration [115, 116]. The dual effects of TZD thus remind us of the coupled regulation of bone homeostasis and energy metabolism by PPAR*γ* [117]. As MAT is a negative regulator of bone, strategies aimed at balancing PPAR*γ* signaling and controlling MAT formation during diabetes treatment might decrease the risk of bone fractures. Fibroblast growth factor 21 (FGF21) is another PPAR*γ* agonist that stimulates glucose uptake in adipocytes and promotes lipid metabolism. It is a promising drug for T2DM, but the side effect also links to bone loss. FGF21 gain-of-function in mice resulted in a striking decrease in bone mass [118]. However, a recent study that treated HFD-induced obesity mice with recombinant human FGF21 (rhFGF21) for 2 weeks did not reach the conclusion of bone loss or any change in mineral apposition rate, bone resorption markers, and bone marrow fat [119]. Therefore, further studies regarding the effect of FGF21 on bone and MAT are needed.

## 5. Outlook

The research over the past years has revealed the important role MAT plays in the bone marrow niche and energy metabolism. The idea that MAT is just a “filler” of the bone marrow is abandoned now. MAT is now recognized as being distinct from peripheral AT in respect of their origin, location, and function, though they still have overlap and interconnection with each other. For example, they coordinated well in energy metabolism via secreted endocrine factors despite their different distribution depots. Interestingly, peripheral AT can achieve “switching” in predetermined settings, but this phenomenon has not been observed in MAT yet. Therefore, future studies clarifying the functional coordination and differences between MAT and extramedullary/peripheral AT are expected. Efforts should also be made to optimize experimental methods to obtain and purify MAT adipocytes. Compared with peripheral AT, MAT in the bone marrow is protected by bone and thus is harder to obtain. Besides, the bone marrow is heterogeneous, as it is composed of marrow stroma, HSCs, MSCs, adipocytes, fibroblasts, and so on. The existence of these cells, especially MSCs/SSCs, which are capable of multilineage differentiation and give rise to cells at different differentiation stages, makes lineage tracing of MAT progenitors difficult. In addition, although several studies have successfully identified SSCs expressing surface markers such as *Osx*, *LepR*, *Nes*, and *CD45^−^CD31^−^Sca^+^CD24^−^* (APCs) that could give rise to adipocyte lineage, the origin of MAT adipocytes has not reached a consensus. Moreover, MAT adipocytes are distributed in different sites, as in the case of cMAT and rMAT. The exact function of these two types of MAT remains unclear. Finally, the relationship between MAT and bone is perplexing. Most studies demonstrated the inverse relationship between MAT and bone. However, there are some studies negating this opinion. For instance, two different mouse strains, C3H/HeJ (C3H) mice and C57BL/6J (B6) mice, exhibit totally distinct MAT and skeletal phenotypes. C3H mice presented both a high level of MAT and bone mineral densities (BMD), while B6 mice exhibited just the opposite, a low level of MAT and BMD [37, 120]. Therefore, more pieces of evidence proving the causative relationship between MAT and bone loss are needed, and based on them, strategies targeting MAT to promote bone remodeling and regeneration are expected.

## Abbreviations

11betaHSD1: 11Beta-hydroxysteroid dehydrogenases
5-FU: 5-Fluorouracil
APCs: Adipogenic progenitor cells
AN: Anorexia nervosa
Beige AT: Beige adipose tissue
BADGE: Bisphenol A diglycidyl ether
BM: Bone marrow
BMSCs: Bone marrow mesenchymal stromal cells
BMP: Bone morphogenetic protein
BAT: Brown adipose tissue
Cebp*α*: CAAT enhancer-binding protein *α*
Cebp*β*: CAAT enhancer-binding protein *β*
CR: Calorie restriction
CMPs: Common myeloid progenitors
cMAT: Constitutive MAT
Dpp4: Dipeptidyl peptidase-4
FA: Fatty acids
FGF21: Fibroblast growth factor 21
FACS: Fluorescence-activated cell sorting
GC: Glucocorticoid
GMPs: Granulocyte-macrophage progenitors
Grem1: Gremlin1
HSCs: Hematopoietic stem cells
HFD: High-fat diet
LepR: Leptin receptor
LSK: Lineage^−^Sca-1^+^CD117^+^
MAT: Marrow adipose tissue
MEPs: Megakaryocyte-erythroid progenitors
MPPs: Multipotent progenitors
Nes: Nestin
OPCs: Osteochondrogenic progenitor cells
Osx: Osterix
PTH1R: Parathyroid hormone receptor 1
PFAS: Performic acid-Schiff staining
PPAR*γ*: Peroxisome proliferated-activated receptor gamma
PRDM16: PRD-BF1-RIZ1 homologous domain containing 16
preAds: Preadipocytes
Rankl: Receptor activator for nuclear factor-*κ*B ligand
rMAT: Regulated MAT
SSCs: Skeletal stem cells
Scf: Stem cell factor
SDF-1: Stromal cell-derived factor 1
SWAT: Subcutaneous WAT
TZD: Thiazolidinediones
T1DM: Type 1 diabetes mellitus
T2DM: Type 2 diabetes mellitus
CGL4: Type 4 congenital generalized lipodystrophy
VWAT: Visceral WAT
WAT: White adipose tissue.
